# Supplementary material for: Scaling of Neuronal Growth and Excitability Through Separable mTORC1 and mTORC2 Pathways
Source: bioRxiv. 2026 Jun 26:2026.06.22.733785. Preprint. [Version 1] doi: 10.64898/2026.06.22.733785 (PMC13320910; doi:10.64898/2026.06.22.733785)
Supplement: Supplement 2 [file NIHPP2026.06.22.733785v1-supplement-2.pdf]

bioRxiv preprint doi: https://doi.org/10.64898/2026.06.22.733785; this version posted June 26, 2026. The copyright holder for this preprint (which was not certified by peer review) is the author/funder, who has granted bioRxiv a license to display the preprint in perpetuity. It is made available under a CC-BY 4.0 International license.

**Figure S1**

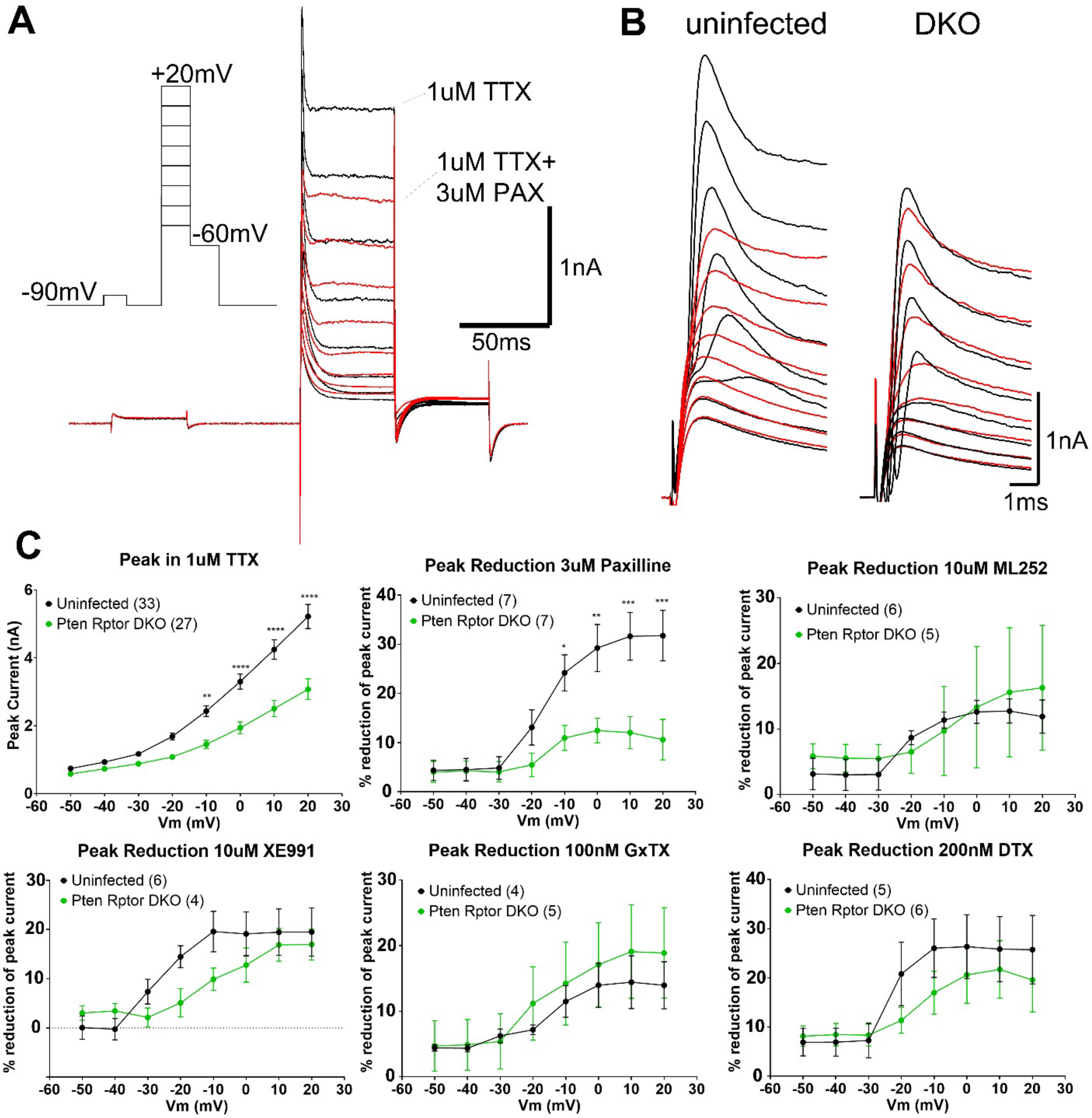

bioRxiv preprint doi: https://doi.org/10.64898/2026.06.22.733785; this version posted June 26, 2026. The copyright holder for this preprint (which was not certified by peer review) is the author/funder, who has granted bioRxiv a license to display the preprint in perpetuity. It is made available under a CC-BY 4.0 International license.

**Figure S1. Voltage-Gated $K^+$ Currents Are Reduced in *Pten;Raptor* dKO and Are Less Sensitive to $K_{Ca}1.1$(BK) Block.**

(A) Granule neurons were voltage-clamped at −90 mV and stepped progressively to +20 mV (protocol inset) to evoke outward $K^+$ currents (black trace). In uninfected wild-type neurons, 3 µM paxilline (PAX) partially blocked the outward current (red trace).

(B) Expanded view of peak $K^+$ currents in uninfected control and Cre-expressing *Pten;Raptor* dKO neurons (black traces). Following application of 3 µM paxilline (red traces), peak current was markedly reduced in uninfected cells but largely unchanged in *Pten;Raptor* dKO neurons (compare pre- and post-paxilline traces).

(C) Peak $K^+$ current amplitude plotted as a function of membrane voltage (Vm) shows reduced current in *Pten;Raptor* dKO neurons (measured in 1 µM TTX). In wild-type neurons, paxilline (3 µM) reduced peak current by ~30%, compared to ~5% in *Pten;Raptor* dKO neurons. This genotype-dependent effect was not observed with the $K_V7$ blockers ML252 (10 µM) or XE991 (10 µM), the $K_V2$ blocker guangxitoxin (GxTx, 100 nM), or the $K_V1$ blocker α-dendrotoxin (DTX, 100 nM). Cell numbers are indicated in parentheses; data are presented as mean ± SEM. (*$p<0.05$, **$p<0.01$, ***$p<0.001$ versus wild-type; mixed-effect model with Sidak's post-hoc).

bioRxiv preprint doi: https://doi.org/10.64898/2026.06.22.733785; this version posted June 26, 2026. The copyright holder for this preprint (which was not certified by peer review) is the author/funder, who has granted bioRxiv a license to display the preprint in perpetuity. It is made available under a CC-BY 4.0 International license.

**Figure S2**

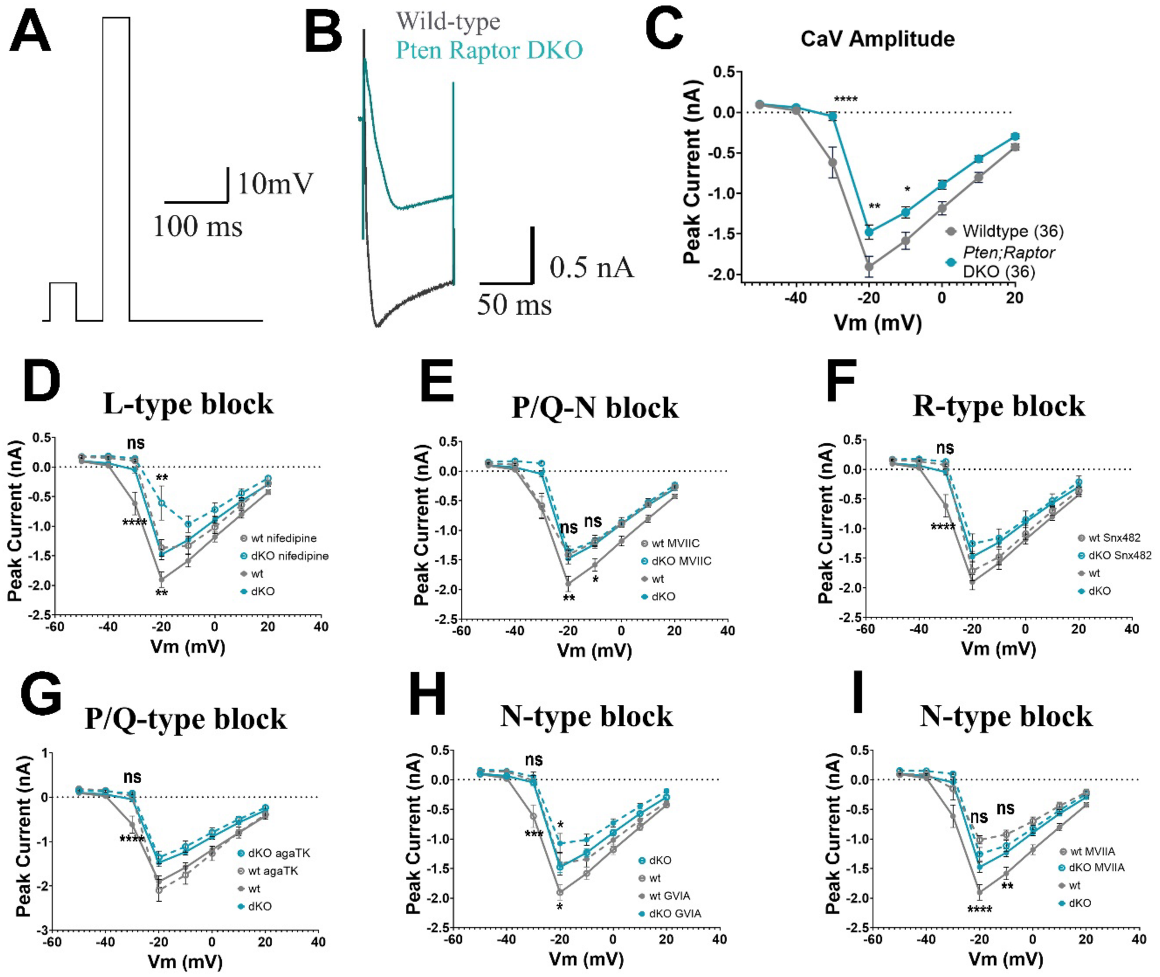

bioRxiv preprint doi: https://doi.org/10.64898/2026.06.22.733785; this version posted June 26, 2026. The copyright holder for this preprint (which was not certified by peer review) is the author/funder, who has granted bioRxiv a license to display the preprint in perpetuity. It is made available under a CC-BY 4.0 International license.

**Figure S2. Voltage-Gated Ca Currents are Reduced in *Pten;Raptor* dKO**

(A) Voltage-clamp protocol for CaV currents. Neurons were held at −90 mV, followed by a 10 mV test pulse and a series of depolarizing steps from −50 to +20 mV. A representative trace from a −20 mV step is shown.

(B) Representative $Ca^{2+}$ current traces from average wild-type (gray) and *Pten;Raptor* dKO (cyan) neurons in response to the −20 mV step.

(C) Current–voltage (I–V) relationship plotting peak $Ca^{2+}$ current amplitude (nA) versus membrane voltage (Vm, mV) for wild-type (gray) and *Pten;Raptor* dKO (cyan) neurons. *Pten;Raptor* dKO neurons exhibit reduced voltage-gated $Ca^{2+}$ currents. n values (neurons) are indicated in parentheses (*p<0.05, **p<0.01, ***p<0.001. ****p<0.0001; mixed-effects model with Šidák multiple-comparisons test).

(D-I)Comparison of $Ca^{2+}$ currents in wild-type (gray) and *Pten;Raptor* dKO (cyan) neurons recorded in the absence (solid lines) or presence (dashed lines) of specific $Ca^{2+}$ channel blockers: L-type blocker nifedipine (10 µM; D), P/Q- and N-type blocker ω-conotoxin MVIIC (1 µM; E), R-type blocker SNX-482 (0.5 µM; F), P/Q-type blocker ω-agatoxin TK (0.2 µM; G), N-type blocker ω-conotoxin GVIA (1 µM; H), and N-type blocker ω-conotoxin MVIIA (50 nM; I). Statistical comparisons below each graph indicate wild-type versus wild-type + blocker; comparisons above indicate *Pten;Raptor* dKO versus *Pten;Raptor* dKO + blocker. (ns p>0.05, *p<0.05, **p<0.01, ***p<0.001. ****p<0.0001 using mixed-effect model with Tukey's multiple comparisons for every condition at ever voltage). If no difference is indicated then there was not a significant effect of that drug on wild-type or *Pten; Raptor* dKO neurons at the indicated voltage.

bioRxiv preprint doi: https://doi.org/10.64898/2026.06.22.733785; this version posted June 26, 2026. The copyright holder for this preprint (which was not certified by peer review) is the author/funder, who has granted bioRxiv a license to display the preprint in perpetuity. It is made available under a CC-BY 4.0 International license.

**Figure S3**

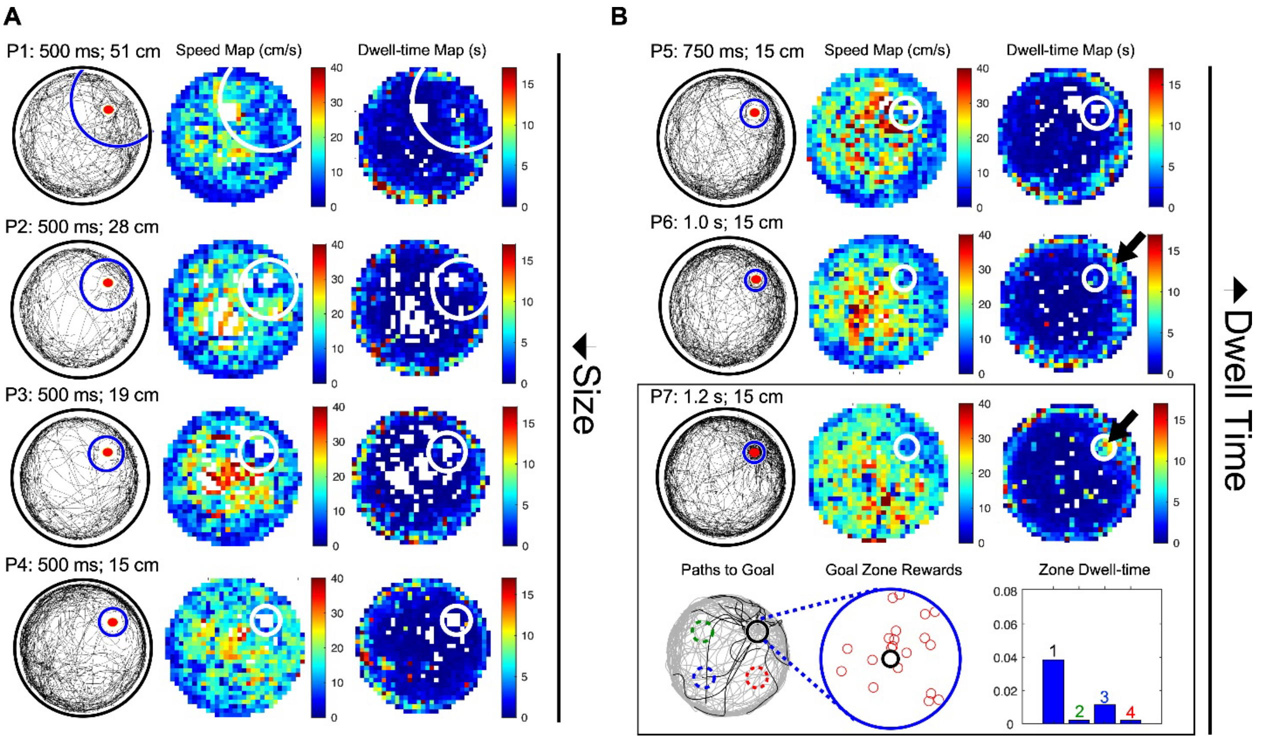

bioRxiv preprint doi: https://doi.org/10.64898/2026.06.22.733785; this version posted June 26, 2026. The copyright holder for this preprint (which was not certified by peer review) is the author/funder, who has granted bioRxiv a license to display the preprint in perpetuity. It is made available under a CC-BY 4.0 International license.

**Figure S3. Object Shaping Paradigm and Performance from a *Pten* KO Mouse**

(A) Left, overall path (black lines), goal zone (blue semicircle/circle), and goal object (red circle); middle, speed map (cm/s); right, dwell time map. Goal zone for a visible object (V1) diameter progressively decreases from 51 cm to 15 cm during phases P1–P4.

(B) Top, required dwell time within V1 increases from 500 ms to 750 ms and 1.2 s during phases P5–P7. Bottom left, rewarded goal entrances (black lines and solid black circle) shown relative to overall path (gray lines) and zones equidistant from the arena center in each quadrant (dashed circles in green, blue, and red). Bottom middle, magnified view of the goal zone showing reward locations. Bottom right, quantification of the proportion of session time spent in zones matching the goal zone diameter (zone 1) and in equidistant control zones in the other three quadrants (zones 2–4). Some *Pten* KO mice required additional sessions to reach criterion due to off-target pauses during early training (black arrow in P6).

bioRxiv preprint doi: https://doi.org/10.64898/2026.06.22.733785; this version posted June 26, 2026. The copyright holder for this preprint (which was not certified by peer review) is the author/funder, who has granted bioRxiv a license to display the preprint in perpetuity. It is made available under a CC-BY 4.0 International license.

**Figure S4**

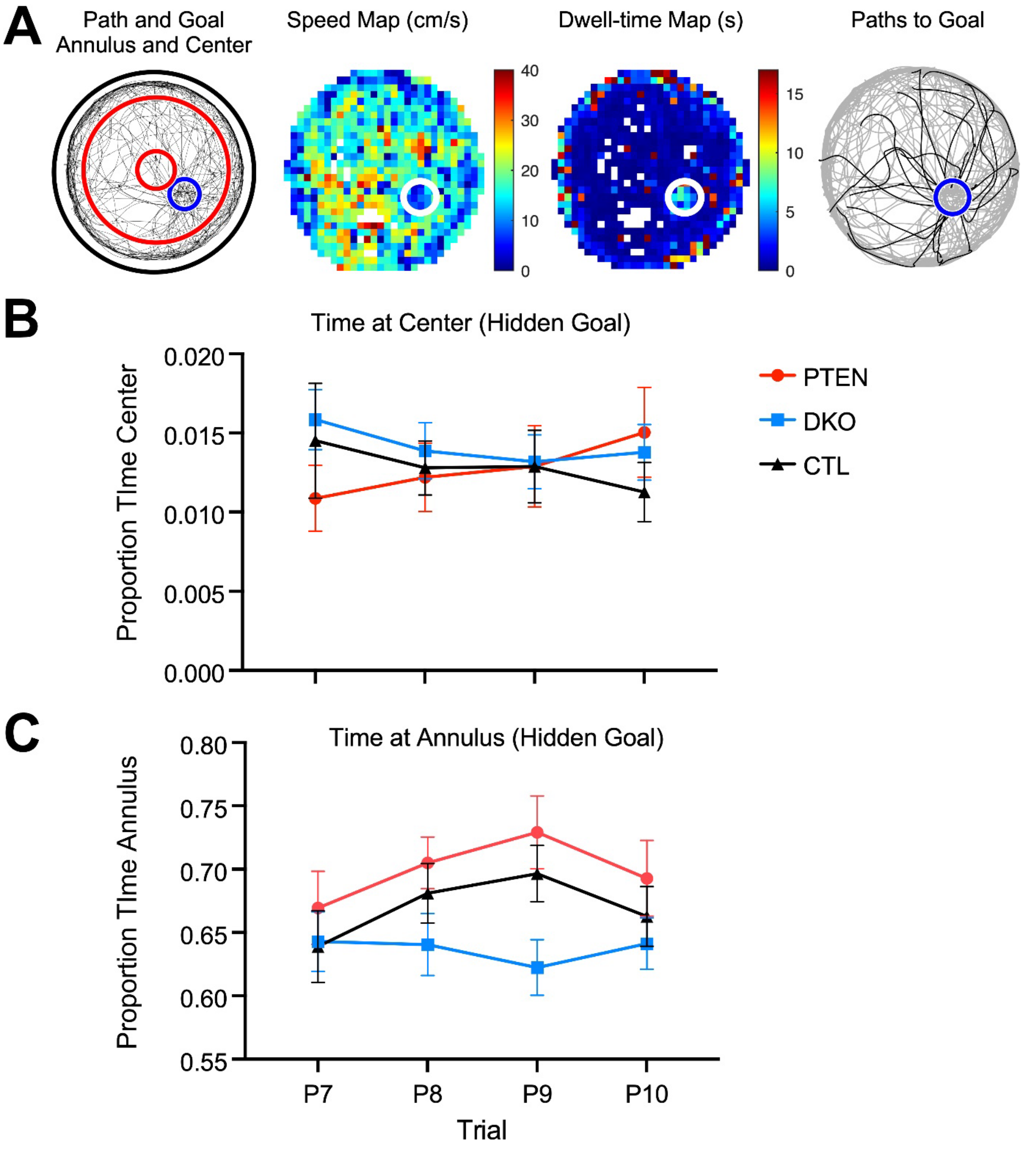

bioRxiv preprint doi: https://doi.org/10.64898/2026.06.22.733785; this version posted June 26, 2026. The copyright holder for this preprint (which was not certified by peer review) is the author/funder, who has granted bioRxiv a license to display the preprint in perpetuity. It is made available under a CC-BY 4.0 International license.

**Figure S4. Spatial cognitive differences in *Pten* KO mice are not due to thigmotaxis:** (A-C) Measurement of the proportion time (A) spent in the cylinder center (B) or annulus (C) shows no significant group x phase interaction effects. Spatial accuracy deficits shown by *Pten* KO during goal rotation sessions are therefore not due to differences in thigmotaxic behavior or increased anxiety levels.

bioRxiv preprint doi: https://doi.org/10.64898/2026.06.22.733785; this version posted June 26, 2026. The copyright holder for this preprint (which was not certified by peer review) is the author/funder, who has granted bioRxiv a license to display the preprint in perpetuity. It is made available under a CC-BY 4.0 International license.

1658 **Figure S5**

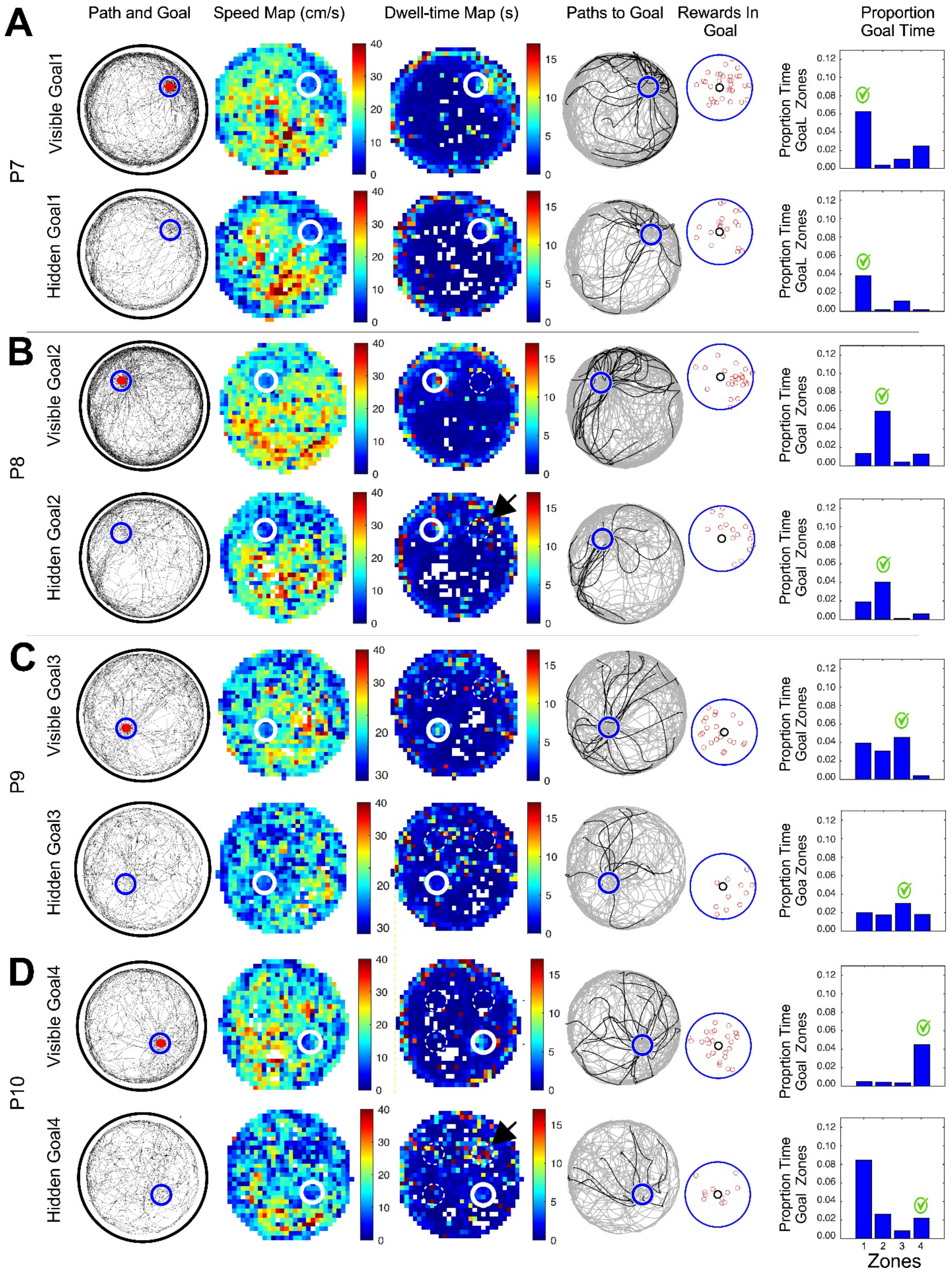

bioRxiv preprint doi: https://doi.org/10.64898/2026.06.22.733785; this version posted June 26, 2026. The copyright holder for this preprint (which was not certified by peer review) is the author/funder, who has granted bioRxiv a license to display the preprint in perpetuity. It is made available under a CC-BY 4.0 International license.

**Figure S5. Example performance of a *Pten* KO mouse in visible (V1-4) and hidden goal (H1-4) probe sessions in phases 7-10.** As in Figure S2, Performance is shown in 6 plots illustrating the overall path and goal location, speed map, dwell-time map, paths to goal, location of rewards within the goal zone, and quantification of the proportion time spent in equal sized zones the same distance from the arena center in each quadrant. Goal-pauses during visible goal probes were generally restricted to the correct goal zone (but see phase 9). Yet in hidden goal sessions, the *Pten* KO mouse tended to keep pausing in zone 1, particularly during Phase 10 when H4 was the correct goal zone.

bioRxiv preprint doi: https://doi.org/10.64898/2026.06.22.733785; this version posted June 26, 2026. The copyright holder for this preprint (which was not certified by peer review) is the author/funder, who has granted bioRxiv a license to display the preprint in perpetuity. It is made available under a CC-BY 4.0 International license.

**Figure S6**

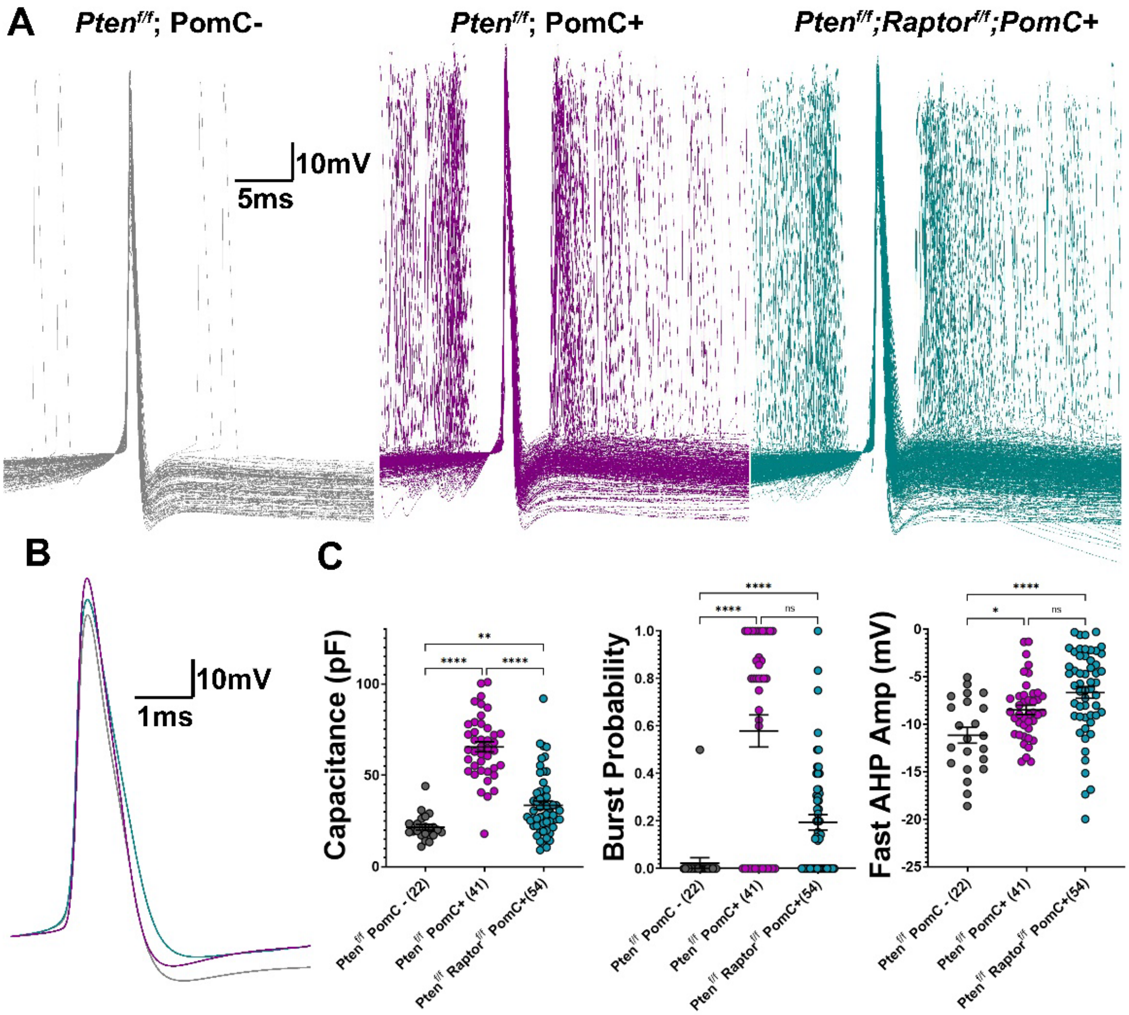

bioRxiv preprint doi: https://doi.org/10.64898/2026.06.22.733785; this version posted June 26, 2026. The copyright holder for this preprint (which was not certified by peer review) is the author/funder, who has granted bioRxiv a license to display the preprint in perpetuity. It is made available under a CC-BY 4.0 International license.

**Figure S6. Whole-Cell Electrophysiology in POMC-Cre *Pten* and *Pten;Raptor* dKO Confirms Bursting Phenotypes Found in Retroviral KO**

(A) Overlay of action potentials recorded at rheobase from *Pten*$^{flx/flx}$;POMC-Cre− (gray), *Pten*$^{flx/flx}$;POMC-Cre+ (magenta), and *Pten*$^{flx/flx}$;*Raptor*$^{flx/flx}$;POMC-Cre+ (cyan) neurons, illustrating prominent burst firing in *Pten* KO and *Pten;Raptor* dKO neurons.

(B) Average action potential waveforms from the same genotypes showing reduced fast afterhyperpolarization (fAHP) amplitude in *Pten* KO and *Pten;Raptor* dKO neurons.

(C) Quantification of membrane capacitance, burst probability, and AHP amplitude across genotypes. (n, is indicated in parentheses; *p<0.05, **p<0.01, ***p<0.001, ****p<0.0001 for capacitance and fAHP a one-way ANOVA with Tukey's post-hoc and for burst probability a fisher's exact test with the contingency of burst vs no burst

bioRxiv preprint doi: https://doi.org/10.64898/2026.06.22.733785; this version posted June 26, 2026. The copyright holder for this preprint (which was not certified by peer review) is the author/funder, who has granted bioRxiv a license to display the preprint in perpetuity. It is made available under a CC-BY 4.0 International license.

**Figure S7**

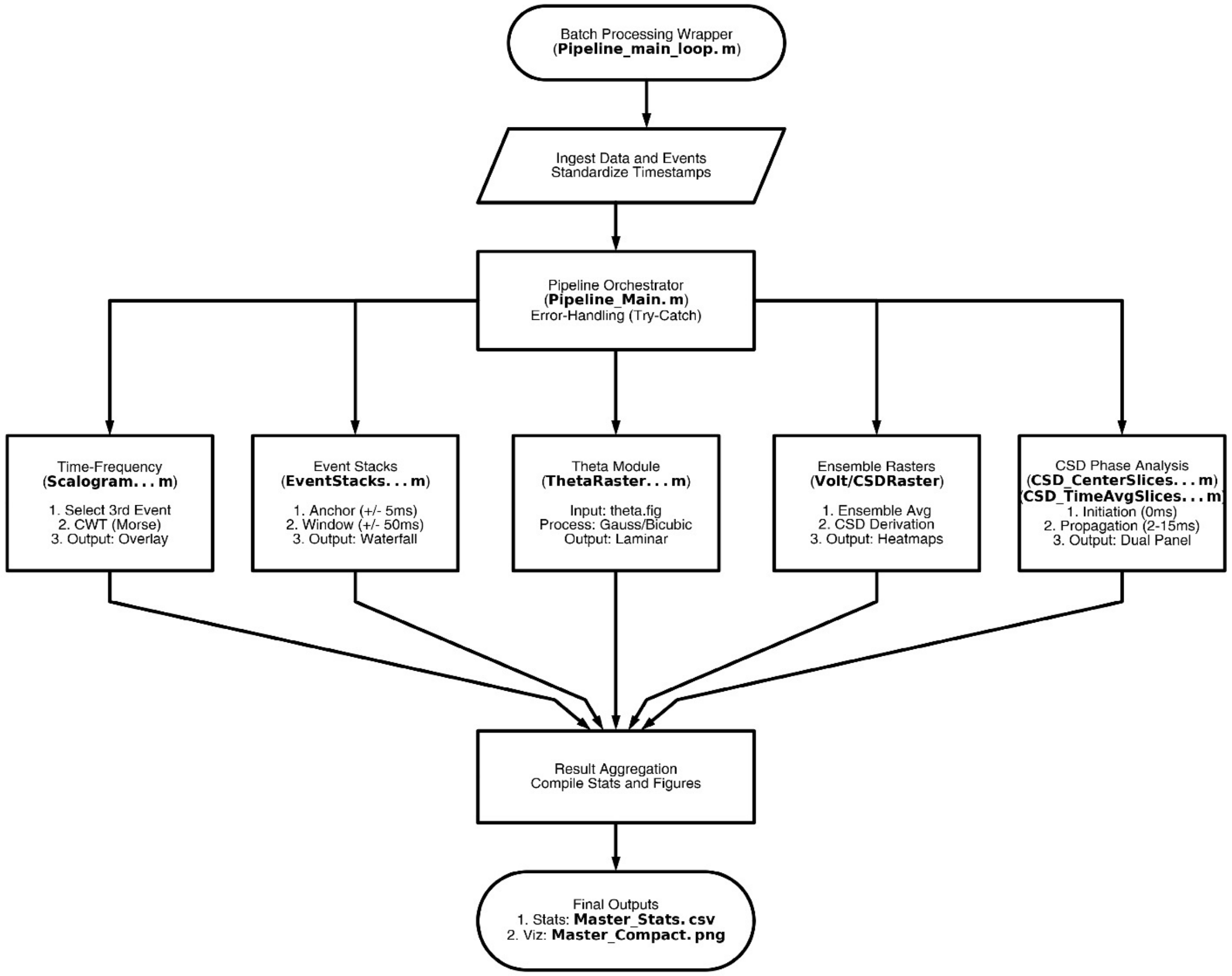

bioRxiv preprint doi: https://doi.org/10.64898/2026.06.22.733785; this version posted June 26, 2026. The copyright holder for this preprint (which was not certified by peer review) is the author/funder, who has granted bioRxiv a license to display the preprint in perpetuity. It is made available under a CC-BY 4.0 International license.

**Figure S7. LFP data analysis pipeline.**
To ensure consistent parameter application and reproducibility across large-scale datasets, all downstream analysis was orchestrated by a custom MATLAB pipeline. A wrapper script managed the iteration through subject directories, ensuring that the same algorithmic standards were applied to every recording session without manual intervention. For each session, the pipeline automatically ingested the pre-processed voltage matrix and the corresponding event timestamp file. Legacy event files were automatically converted to a standardized tabular format prior to analysis. The pipeline sequentially triggered seven independent analytical modules (Theta/Waveform Raster, Event Triggered Waveform Stacks, Voltage Raster, CSD Raster, CSD Center Slices Initiation Phase, CSD Time-Averaged Slices Propagation Phase, and Time-Frequency Scalograms). Each module operated within a robust error-handling framework to prevent localized artifacts from halting the batch processing of an entire dataset Following analysis, statistical outputs (e.g., peak amplitudes, half-widths) from all modules were aggregated into a master CSV file and summary visualizations were compiled into high-resolution figure panels.

bioRxiv preprint doi: https://doi.org/10.64898/2026.06.22.733785; this version posted June 26, 2026. The copyright holder for this preprint (which was not certified by peer review) is the author/funder, who has granted bioRxiv a license to display the preprint in perpetuity. It is made available under a CC-BY 4.0 International license.

**Figure S8**

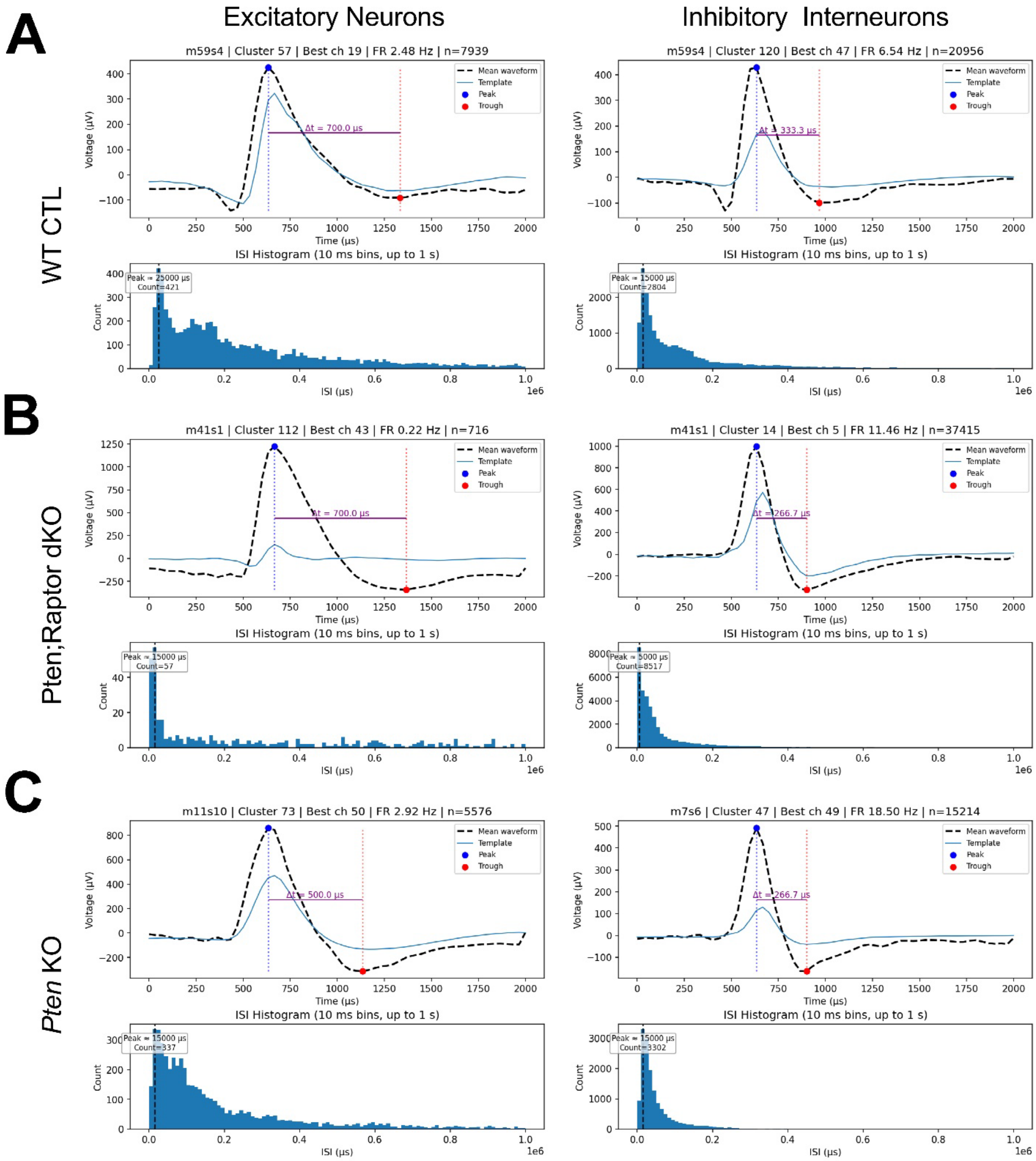

bioRxiv preprint doi: https://doi.org/10.64898/2026.06.22.733785; this version posted June 26, 2026. The copyright holder for this preprint (which was not certified by peer review) is the author/funder, who has granted bioRxiv a license to display the preprint in perpetuity. It is made available under a CC-BY 4.0 International license.

**Figure S8. Example Kilosort clustering.**

(A-C). Classification of excitatory (left) and inhibitory (right) neurons based on extracellular waveform properties. Kilosort was used for automated spike sorting and clustering, followed by analysis of waveform features including spike duration and inter-spike interval (ISI) for wild-type (A), *Pten;Raptor* dKO (B), and *Pten* KO (C) mice.
